# Patterns and trends of mortality from metastatic colorectal cancer in Shanghai, China from 2005 to 2021: a population-based retrospective analysis

**DOI:** 10.1007/s00432-023-05518-z

**Published:** 2024-02-02

**Authors:** Xuelin Cheng, Jing Zhou, Yichen Chen, Yajun Zhao, Huichao Zheng, Qizhe Wang, Xiaopan Li, Sunfang Jiang

**Affiliations:** 1 Department of Health Management Center, Zhongshan Hospital, Fudan University, Shanghai, China; 2 Department of General Practice, Zhongshan Hospital, Fudan University, Shanghai, China; 3 Office of Scientific Research and Information Management, Pudong Institute of Preventive Medicine, Pudong New Area, Shanghai, China

**Keywords:** Metastatic colorectal cancer, Population aging, Mortality, Year of life lost, Trend analysis

## Abstract

**Purpose:**

Metastatic colorectal cancer (mCRC) is the leading cause of CRC deaths, however, the relative epidemiological research was insufficient. We aimed to analyze the patterns and trends of mortality of mCRC in Shanghai with a more complete system for monitoring the cause of death of the population and find potential methods to reduce the burden of CRC in China.

**Methods:**

Mortality data from 2005 to 2021 of mCRC deaths were obtained from the mortality registration system in Shanghai. We analyzed the crude mortality rates, age-standardized mortality rates, and rates of years of life lost (YLL rates) of mCRC. In addition, the trends were quantified using Joinpoint Regression software.

**Results:**

A total of 4,386 mCRC deaths were included, with 1,937 (44.16%) liver metastases and 1,061 (24.19%) lung metastases. The crude mortality rate and age-standardized mortality rate of mCRC were 9.09 per 10^5^ person-years and 3.78 per 10^5^ person-years, respectively. The YLL was 50,533.13 years, and the YLL rate was 104.67 per 10^5^ person-years. The overall annual crude mortality rate of mCRC increased by 1.47% (95% CI 0.28–2.68%, *P* < 0.001) from 2005 to 2021. The crude mortality rate of mCRC increased by 3.20% per year (95% CI 1.80–4.70%, *P* < 0.001) from 2005 to 2013, but the trend of mortality growth remained stable from 2013 to 2021. The YLL rates remained stable between 2005 and 2021.

**Conclusions:**

Population aging was the most likely factor responsible for the increase in CRC mortality in Pudong. Physical examinations and screenings for the elderly were possible reasons for reducing the burden of CRC in fast-growing regions.

**Supplementary Information:**

The online version contains supplementary material available at 10.1007/s00432-023-05518-z.

## Introduction

In recent years, the incidence and mortality of colorectal cancer (CRC) have been increasing in developing countries because of lifestyle changes (e.g., diet), especially in China (Chen et al. 2021a, b). The estimated incidence and mortality rate of CRC in 2022 were about 29.51 and 14.14 per 10^5^ person-years, thus being among the top 5 causes of cancer deaths in China (Zheng et al. 2022). Metastatic CRC (mCRC) is the leading cause of CRC deaths—approximately 45% of patients suffering from CRC will experience metastases later resulting in a relatively high overall mortality rate (Schmoll et al. 2012), and the liver is the most common site of metastases worldwide (Kuchel et al. 2021). The proportion of rectal cancer cases in China was reportedly higher than in Western countries, where the transfer of malignant cells to the lungs is more common. Therefore, patients with lung metastases are also common in China (Suthananthan et al. 2018; Association 2019). In addition, there is a lack of relevant epidemiological data on other metastatic sites (such as bone, brain, and ovary) in China.

The latest data from the National Cancer Center (NCC) show that CRC was the second most common cancer and the fourth most common cause of cancer deaths in China (Zheng et al. 2022). Although the burden of CRC in China is heavy, epidemiological evidence of mCRC is insufficient. However, knowing the patterns and trends of mCRC deaths is of great significance in providing excellent support for evidence-based medicine for formulating relevant prevention and screening policies in the future.

Pudong, with 3.22 million permanent residents, is the largest district in Shanghai, China, and accounts for 20% of the total population in Shanghai. The national government has strongly developed Pudong since 1990, making it the fastest-growing area in Shanghai and the country. Furthermore, Pudong is the only district in Shanghai with towns and villages (Li et al. 2018). With the increasing development and globalization of the China economy, people’s lifestyle behaviors are becoming increasingly Westernized, especially in economically developed areas such as Pudong.

The objective of this study was to investigate the patterns and trends of mCRC deaths in rapidly developing cities such as Shanghai to provide substantial evidence-based medical evidence for policymakers in the future.

## Methods

This retrospective population-based study was conducted following the 2000 Declaration of Helsinki. The protocol of the present study was approved by the ethics committee of the Shanghai Pudong New Area Center for Disease Control and Prevention (IRB2016-04-0586). However, due to the retrospective and anonymous characteristics, informed consent from each participant was waived.

### Data source

The population-based mCRC mortality data of permanent residents from 2005 to 2021 were collected by the mortality registration system in Shanghai, which has been used since the beginning of the twenty-first century. All mCRC deaths of permanent residents were identified without age restriction. According to the International Classification of Diseases 10th version (ICD-10), C18–C21 refers to colorectal cancer, C78.0 refers to secondary malignant neoplasm of the lung, C78.7 refers to secondary malignant neoplasm of the liver, C79.5 refers to secondary malignant neoplasm of bone and bone marrow, C78.6 refers to secondary malignant neoplasm of retroperitoneum and peritoneum, C78.8 refers to secondary malignant neoplasm of other and unspecified digestive organs, C79.3 refers to secondary malignant neoplasm of brain and cerebral meninges, C79.1 refers to secondary malignant neoplasm of bladder and other and unspecified urinary organs, C79.6 refers to secondary malignant neoplasm of ovary, C78.2 refers to secondary malignant neoplasm of pleura, C79.0 refers to secondary malignant neoplasm of kidney and renal pelvis, C79.2 refers to secondary malignant neoplasm of skin, C78.3 refers to secondary malignant neoplasm of other and unspecified respiratory organs, and C79.7 refers to secondary malignant neoplasm of adrenal gland. The cause of death was coded by clinicians and checked by the local Center for Disease Control and Prevention (CDC).

### Variables and measurement

The Public Security Bureau and the Statistics Bureau provided the complete data of the included participants. The mortality from mCRC was measured using the crude mortality rate and the age-standardized mortality rate. The crude mortality rates (per 10^5^ person-year) were calculated as the total number of mCRC deaths each year divided by the corresponding annual average population in Pudong and shown as per 10^5^ persons. Age-standardized mortality rates (per 10^5^ person-year) by Segi’s world standard population were calculated. The premature deaths of mCRC were measured by the years of life lost (YLL) and YLL rate, which was defined as the mCRC deaths that occurred before the average age of death in Shanghai (around 82 years) (Peng et al. 2021).

We performed separate statistical analyses to determine the quantity and proportion of metastases in different sites of colorectal cancer. Furthermore, we will place particular emphasis on the examination of age at death, mortality, and YLL specifically related to liver metastases and lung metastases.

### Statistical analysis

Age-specific mortality rates and YLLs were analyzed using the following age groups: 0–29 years, 30–44 years, 45–59 years, 60–69 years, 70–79 years, and ≥ 80 years. The mortality and YLL rate trends were analyzed using Joinpoint Regression software (version 4.9.1.0; NCI) and described as increasing or decreasing when the annual percent change was statistically significant (based on a two-sided *p* value < 0.05); otherwise, they were described as stable. The *Z*-test was used to assess whether the APC was statistically different from zero.

## Results

### Patients and characteristics

**Fig. 1:**
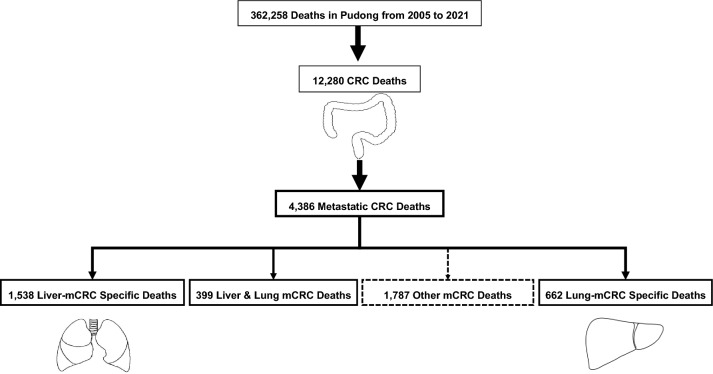
Flowchart of selected deaths from total deaths in Pudong, Shanghai during 2005–2021

Of 12,280 CRC deaths during 2005–2021, 4386 (35.72%) residents died of mCRC, including 1538 deaths (1538/4386, 35.07%) due to liver-specific metastases, 662 deaths (662/4386, 15.09%) due to lung-specific metastases, and 399 deaths (399/4386, 9.10%) due to simultaneous liver and lung metastases (Fig. 1). Among the 4386 patients, the median age of death was 71.30 (95% CI 24.68–99.20) years and 72.69 (95% CI 22.74–99.09) years for males and females, respectively (Table 1).

**Table 1:** Baseline characteristics of the people who died of mCRC during 2005–2021, along with different metastatic sites

Characteristic	Deaths (*n*, %)	Age at death (median)	Age at death (range)	CMR (/10^5^)	ASMRW (/10^5^)	YLL (years)	YLL rate (/10^5^)
Total	4386 (100.00%)	71.81	22.74–99.20	9.09	3.78	50,533.13	104.67
Male	2621 (59.76%)	71.30	24.68–99.20	10.88	4.78	29,105.37	120.83
Female	1765 (40.24%)	72.69	22.74–99.09	7.30	2.89	21,427.75	88.59
Metastatic sites							
Lung^a^	662 (15.09%)	74.86	32.72–98.15	1.37	0.53	6843.37	14.18
Male	400 (9.12%)	74.80	32.72–98.14	1.74	0.72	4203.21	17.45
Female	262 (5.97%)	75.06	43.67–91.41	1.01	0.37	2640.16	10.92
Liver^b^	1538 (35.07%)	70.97	24.68–99.20	3.19	1.34	17,889.89	37.06
Male	925 (21.10%)	71.26	25.58–99.20	4.06	1.80	11,122.45	46.17
Female	613 (13.98)	70.60	24.68–97.01	2.32	0.91	6767.44	27.98

*ASMRW* age-standardized mortality rate by Segi’s world standard population, *CMR* crude mortality rate, *YLL* years of life lost

^a^Lung-specific metastases

^b^Liver-specific metastases

### Mortality

The crude mortality rate of mCRC was 9.09 per 10^5^ person-years in the whole cohort, with 10.88 per 10^5^ person-years in males and 7.30 per 10^5^ person-years in females. The crude mortality rate of liver-specific metastases (3.19 per 10^5^ person-years) was higher than that of lung-specific metastases (1.37 per 10^5^ person-years). The age-standardized mortality rate of mCRC was 3.78 per 10^5^ person-years for all 4386 patients. In addition, the age-standardized mortality rate was 4.78 per 10^5^ person-years in males, which was higher than in females (2.89 per 10^5^ person-years). Furthermore, the age-standardized mortality rate of liver-specific metastases (1.34 per 10^5^ person-years) was higher than that of lung-specific metastases (0.53 per 10^5^ person-years) (Table 1).

### Premature deaths

The YLL was 50,533.13 years for the whole cohort from 2005 through 2021, with the YLL rate of 104.67 per 10^5^ person-years; both were higher in males than females (YLL: 29,105.37 years vs. 21,427.75 years) (YLL rate: 120.83 per 10^5^ person-years vs. 88.59 per 10^5^ person-years). The YLL of liver metastases was 17,889.89 years, with a YLL rate of 37.06 per 10^5^ person-years, which was higher than lung metastases (YLL: 6843.37 years; YLL rate: 14.18 per 10^5^ person-years) (Table 1).

### Age disparity

The three age groups with the highest crude mortality rates were aged ≥ 80 years (52.61 per 10^5^ person-years), 70–79 years (37.51 per 10^5^ person-years), and 60–69 years (16.99 per 10^5^ person-years), respectively. Moreover, the three age groups with the highest YLL rates were aged 70–79 years (349.73 per 10^5^ person-years), ≥ 80 years (280.39 per 10^5^ person-years), and 60–69 years (239.87 per 10^5^ person-years), respectively. Around 80.35% of deaths caused by mCRC occurred among individuals aged 60 years and above. Regardless of age group, the crude mortality rates, YLL, and YLL rates associated with liver metastases were consistently higher than those associated with lung metastases (Supplementary Table S1).

### Primary location and metastatic sites

The primary locations of mCRC were the colon (C18, 59.26%), rectum (C20, 38.62%), the rectosigmoid junction (C19, 1.69%), and anus/anal canal (C21, 0.43%), respectively (Table S2). The more common metastatic sites were the bone and bone marrow (C79.5, 4.03%), retroperitoneum and peritoneum (C78.6, 3.64%), and brain and cerebral meninges (C79.3, 2.41%) (Table S2), second only to the liver (C78.7, 44.16%) and lungs (C78.0, 24.19%).

### Multimorbidity

**Fig. 2:**
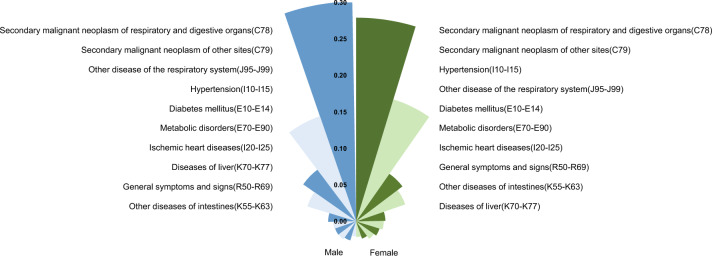
The status of multimorbidities in mCRC deaths

The five most common comorbidities of both male and female patients with mCRC were other diseases of the respiratory system (J95–J99), hypertension (I10–I15), diabetes mellitus (E10–E14), metabolic disorders (E70–E90), and ischemic heart diseases (I20–I25) (Fig. 2).

### Trends

**Fig. 3:**
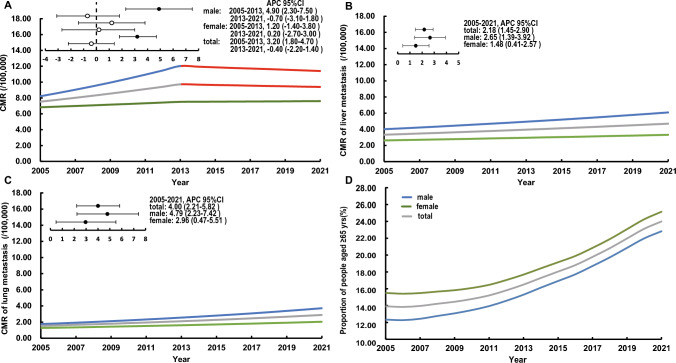
**A** Increased CMR in people who died of mCRC during 2005–2021. **B** Increased CMR in people who died of liver metastases. **C** Increased CMR in people who died of lung metastases. **D** The proportion of deaths from mCRC aged ≥ 65 years during 2005–2021 in Pudong, Shanghai, China

The overall annual crude mortality rate of mCRC increased by 1.47% (95% CI 0.28–2.68%; *P* < 0.001) from 2005 to 2021. The crude mortality rate of mCRC increased by 3.20% per year (95% CI 1.80–4.70%; *P* < 0.001) from 2005 to 2013, however, the trend of mortality growth remained stable from 2013 to 2021 (Fig. 3A). The overall annual crude mortality rate of liver metastases increased by 2.18% (95% CI 1.45–2.90%; *P* < 0.005) during the 16 years, as well as 4.00% (95% CI 2.21–5.82%) in lung metastases, which was higher than the former (Fig. 3B, C). In addition, the proportion of mCRC patients aged ≥ 65 years continued to grow from 2005 to 2021 (Fig. 3D).

The age-standardized mortality rate of mCRC decreased by -1.23% (95% CI − 1.87 to − 0.58%; *P* < 0.001), especially in female patients (− 2.02%; 95% CI − 3.03 to − 1.00%; *P* < 0.001). The age-standardized mortality rates of the group aged 0–29 years, aged 30–44 years, aged 45–59 years, and the group aged ≥ 80 years were stable, however, the age-standardized mortality rates of the group aged 60–69 years and those aged 70–79 decreased by − 1.67% (*P* < 0.001).

**Fig. 4:**
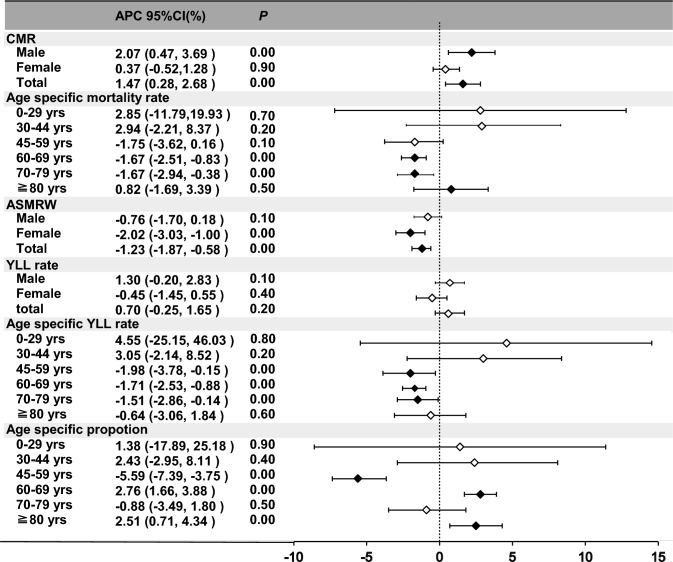
Trends in CMRs, ASMRWs, age-specific proportion of mortality, and YLL of people who died of mCRC according to sex and age group in Shanghai, Pudong, China during 2005–2021. *CMR* crude mortality rate (per 10^5^ person-year), *ASMRW* age-standardized mortality rate by Segi’s world standard population (per 10^5^ person-year), *YLL* years of life lost, *APC* annual percentage change, *CI* confidence interval

The overall YLL rates remained stable between 2005 and 2021. The YLL rates of the group aged 0–29 years, aged 30–44 years, and the group aged ≥ 80 years were stable. The YLL rates of groups aged 45–59 years, 60–69 years, and 70–79 years decreased separately by − 1.98% (95% CI − 3.78 to − 0.15%; *P* < 0.001), − 1.71% (95% CI − 2.53 to − 0.88%; *P* < 0.001), and − 1.51% (95% CI − 2.86 to − 0.14%; *P* < 0.001) per year (Fig. 4).

## Discussion

In the present study, we found that the mortality for mCRC increased continuously in Pudong from 2005 to 2021. However, during the same period, the age-standardized mortality rate declined. The trend of mortality growth remained stable from 2013 to 2021. The YLL rates for mCRC increased with advancing age, peaking in the 70–79-year age group. Among the patients who died of mCRC, colon cancer, and rectal cancer accounted for 59.26% and 38.62% of cases, respectively, similar to a multicenter retrospective epidemiological survey in which the proportion of colon cancer was about 60%, while rectal cancer was 40% in China (Shi et al. 2021). In addition, in common with other types of cancers, there was a striking difference between men and women: men had a much higher mCRC mortality than women.

Mortality from mCRC in Pudong increased during the study period. However, a stable trend has been observed since 2013, which may be due to the physical examinations and the introduction of a CRC screening project. The free physical examinations for the elderly as a basic public health program began in 2009 (Commission 2009). In 2013, a national cancer screening project was also launched, with Shanghai being one of the first cities to participate (Chen et al. 2019; Wu et al. 2019). The target demographic for this project comprised residents between the ages of 50 and 74 years, and as of the end of 2016, two rounds of screenings had already been concluded. The participants undergoing screening would undergo immunochemical fecal occult blood testing and risk assessment. For individuals who receive positive screening results, further colonoscopy examinations would be conducted. Of the detected CRC through screening, 51.6% were found at an early stage, which was significantly higher than the current diagnosis rate of early-stage CRC in our country, which does not exceed 10% (Gong et al. 2018). The findings indicated that the detection rate of CRC and the 5-year survival rate among individuals who participated in the screening program significantly surpassed those of individuals who did not participate in the program (Li et al. 2019). Some research also has suggested that screening may reduce the mortality of mCRC and alleviate the disease burden of mCRC (Chen et al. 2019). Indeed, screening may be critical for CRC, especially in high-risk areas such as Shanghai, one of the fastest growing cities in China, with a rising incidence of CRC, possibly related to population aging and increasing consumption of a Western diet (Chen et al. 2021a, b; Zhong et al. 2022).

The advancements in treatment modalities also have a positive impact on the mortality rate of mCRC. For mCRC cases where the cancer has only spread to the liver or lungs, surgical intervention enables the complete removal of the lesions. However, for unresectable mCRC, systemic chemotherapy serves as the primary therapeutic approach, and various studies have suggested that intensive treatment with multiple systemic therapies can enhance the survival of individuals with mCRC by a span of 2–3 years (Heinemann et al. 2014). Moreover, with the progressive advances in molecular profiling, clinical practitioners are empowered to design personalized treatment regimens, effectively prolonging the survival period of individuals diagnosed with mCRC (Biller and Schrag 2021).

This study found that mCRC mortality was related to advanced age in Pudong, which is the area in Shanghai with the largest elderly population. Being one of the cancers associated with aging, CRC is profoundly influenced by the effect of population aging on its mortality rate, which cannot be overlooked. The proportion of the population aged 65 years and older in China increased during 2004–2017, from 8.55 to 11.11% in urban areas, and the mortality rate for aging-related cancers increased by 1.52 person-years. The proportion of elderly individuals also increased from 7.53 to 11.61% in rural areas and the mortality rate for aging-related cancers increased by 3.21 person-years (Liu et al. 2016; Laconi et al. 2020; Chen et al. 2022a, b). According to the 2020 census data, as of November 2020, the total population of Shanghai was approximately 24.87 million people, with individuals aged 65 and above constituting 16.28% of the total population. Mortality rates are growing faster because of population aging (Cardoso et al. 2022), especially in people older than 80 years. To mitigate the current population’s aging trend, China introduced a two-child or even a three-child policy (instead of the former longstanding one-child policy) and delayed the retirement age. However, the effects of the new policy on rapid population aging will not be evident for 2 decades (Zeng and Hesketh 2016).

In this study, we found that the most common metastatic sites from CRC were the liver and lung, which is consistent with previous reports (Dave et al. 2015). However, the mortality and YLL rates of patients with liver-only metastases were significantly higher than those with lung-only metastases, which may be associated with different treatment technologies and tumor microenvironments (Zhang et al. 2020; Chandra et al. 2021). Surgical resection and chemotherapy are the optimal treatments for mCRC, but the difficulty of surgery and the response rates to chemotherapy can vary with the metastases of different sites (Nordlinger et al. 2009). We paid attention to CRC lung metastases because the proportion of rectal cancers that tend to transfer to the lung in China is higher than in some developed countries (50% vs. 30%) (Wu et al. 2019). However, the mechanisms for lung metastases of CRC are still not well studied (Shen et al. 2021). Lung resection is recognized as the standard treatment for resectable lung metastases, yet a considerable body of research suggested that surgical resection did not exhibit discernible improvement in the survival rate of patients (Siebenhüner et al. 2020; Zhang et al. 2020). The latest advancements in chemotherapy have demonstrated remarkable enhancement in the therapeutic efficacy of distant metastases, thereby indicating the opportune moment to reevaluate the significance of pulmonary resection (Ogawa et al. 2021).

We also found a significant disparity in mortality and YLL rates between men and women, perhaps due to lifestyle differences: it is widely known that men smoke and drink more, have higher levels of work pressure, and get less sleep by staying up later than women (Pantell et al. 2013). In addition, a study about gender disparity in cancer suggested that men have a higher risk of cancer than women at most shared anatomic sites (Jackson et al. 2022). Therefore, colorectal cancer risk prediction models may incorporate male sex as a significant risk factor.

In this study, the three most common chronic comorbidities in mCRC patients were respiratory diseases, hypertension, and diabetes. Respiratory diseases are common in mCRC, possibly due to smoking (Jang et al. 2020; Alwers et al. 2021). Indeed, it is widely established that smoking is strongly associated with many types of cancer. Moreover, it is worth noting that women in Asia are commonly exposed to related risk factors, such as environmental smoke, secondhand tobacco smoke, and cooking fumes (McGee et al. 2019; Vermeulen et al. 2019; Larsson et al. 2020; Li et al. 2020; Wen et al. 2022; Yeo et al. 2022). Chemotherapy is the leading treatment for mCRC, but hypertension is one of the main side effects (Berger et al. 2017; Van Cutsem et al. 2019). Research suggests that hypertension is related to the effect of chemotherapy for mCRC (Sibertin-Blanc et al. 2015), and reasonable control of blood pressure levels can improve the efficacy of chemotherapy drugs and the clinical prognosis of patients with mCRC (Shen et al. 2020; Lombardi et al. 2022). Therefore, good control of hypertension can improve the management of mCRC. Finally, the risk of CRC is higher in diabetic patients, and type 2 diabetes is an adverse prognostic factor for survival in CRC (Singh et al. 2016; Ottaiano et al. 2022). Some studies have shown that a genetic mutation in mCRC patients causes them to benefit from metformin treatment, but not insulin treatment (Pradhan et al. 2020; Xie et al. 2020).

This study has some limitations. First, we did not have enough data to extract the time of metastases. Our initial diagnosis time was often when the patients had developed relatively advanced CRC symptoms. The participation rate in screening high-risk populations was low, mainly due to the high inspection cost and improper screening strategies (Chen et al. 2019). Second, although Shanghai has the most advanced mortality registration system in China, the recorded cause of death may not be reliable for deaths that occur at home. However, some researchers have performed a more standardized diagnosis routine of causes of a home death and found that this did not improve the accuracy of the available results significantly, with a great improvement in underdeveloped areas (Chen et al. 2022a, b). Finally, as our study was the first to report premature death of mCRC, we cannot compare our findings with those of other studies. Nevertheless, metastatic cancer is the leading cause of CRC deaths, and our study can provide some data for clinicians and policymakers so that they can promote early screening for colorectal cancer and enhance the treatment of comorbidities.

## Supplementary Information

Below is the link to the electronic supplementary material.

Supplementary file1 (DOCX 20 KB)

Supplementary file2 (DOCX 17 KB)

## Data Availability

The data that support the fndings of this study are available on request from the corresponding author, upon reasonable request.
