# Supplementary material for: Patterns and trends of mortality from metastatic colorectal cancer in Shanghai, China from 2005 to 2021: a population-based retrospective analysis
Source: J Cancer Res Clin Oncol. 2024 Feb 2;150(2):68. doi: 10.1007/s00432-023-05518-z (PMC10837271; doi:10.1007/s00432-023-05518-z)
Supplement: Supplementary file 1 — Supplementary file1 (DOCX 20 KB) [file 432_2023_5518_MOESM1_ESM.docx]

| Table S1-1 \| Age-specific mortality and burden of total mCRC during 2005-2021. | | | | | |
| --- | --- | --- | --- | --- | --- |
| Age group  (years) | Deaths  (N) | Proportion  (%) | CMR (/10^5^) | YLL (years) | YLL rate (/10^5^) |
| 0-29 | 12 | 0.27 | 0.16 | 322.80 | 2.57 |
| 30-44 | 99 | 2.26 | 0.90 | 2364.93 | 21.59 |
| 45-59 | 706 | 16.10 | 5.72 | 13324.30 | 107.94 |
| 60-69 | 1153 | 26.29 | 16.99 | 16275.52 | 239.87 |
| 70-79 | 1344 | 30.64 | 37.51 | 12532.20 | 349.73 |
| ≥80 | 1072 | 24.44 | 52.61 | 5713.37 | 280.39 |
| Total | 4386 | 100.00 | 9.09 | 50533.13 | 104.67 |
| ASMRW, age-standardized mortality rate by Segi’s world standard population; CMR, crude mortality rate; YLL, years of life lost. | | | | | |
|  |  |  |  |  |  |
| Table S1-2 \| Age-specific mortality and burden of total lung-mCRC during 2005-2021. | | | | | |
| Age group (years) | Deaths  (N) | Proportion  (%) | CMR (/10^5^) | YLL (years) | YLL rate (/10^5^) |
| 0-29 | 0 | 0.00 | 0.00 | 0.00 | 0.00 |
| 30-44 | 16 | 1.51 | 0.15 | 386.07 | 3.52 |
| 45-59 | 140 | 13.20 | 1.13 | 2650.23 | 21.47 |
| 60-69 | 260 | 24.51 | 3.83 | 3624.36 | 53.42 |
| 70-79 | 343 | 32.33 | 9.57 | 3184.42 | 88.86 |
| ≥80 | 302 | 28.46 | 14.82 | 1566.82 | 76.89 |
| Total | 1061 | 100.00 | 2.20 | 11411.90 | 23.64 |
| ASMRW, age-standardized mortality rate by Segi’s world standard population; CMR, crude mortality rate; YLL, years of life lost. | | | | | |
|  |  |  |  |  |  |
| Table S1-3 \| Age-specific mortality and burden of total liver-mCRC during 2005-2021. | | | | | |
| Age group (years) | Deaths  (N) | Proportion  (%) | CMR (/10^5^) | YLL (years) | YLL rate (/10^5^) |
| 0-29 | 5 | 0.26 | 0.07 | 134.24 | 1.07 |
| 30-44 | 45 | 2.32 | 0.41 | 1072.44 | 9.79 |
| 45-59 | 307 | 15.85 | 2.49 | 5736.55 | 46.47 |
| 60-69 | 550 | 28.39 | 8.11 | 7736.91 | 114.03 |
| 70-79 | 578 | 29.84 | 16.13 | 5392.63 | 150.49 |
| ≥80 | 452 | 23.34 | 22.18 | 2385.65 | 117.08 |
| Total | 1937 | 100.00 | 4.01 | 22458.42 | 46.52 |
| ASMRW, age-standardized mortality rate by Segi’s world standard population; CMR, crude mortality rate; YLL, years of life lost. | | | | | |
