# Supplementary material for: Patterns and trends of mortality from metastatic colorectal cancer in Shanghai, China from 2005 to 2021: a population-based retrospective analysis
Source: J Cancer Res Clin Oncol. 2024 Feb 2;150(2):68. doi: 10.1007/s00432-023-05518-z (PMC10837271; doi:10.1007/s00432-023-05518-z)
Supplement: Supplementary file 2 — Supplementary file2 (DOCX 17 KB) [file 432_2023_5518_MOESM2_ESM.docx]

**Table S2.** The proportion of different primary locations and metastatic sites of colorectal cancer

| **ICD-10** | **Disease** | **Deaths (n)** | **Proportion (%)** |
| --- | --- | --- | --- |
| **Primary location** | |  |  |
| **C18** | Malignant neoplasm of colon | 2599 | 59.26% |
| **C20** | Malignant neoplasm of rectum | 1694 | 38.62% |
| **C19** | Malignant neoplasm of rectosigmoid junction | 74 | 1.69% |
| **C21** | Malignant neoplasm of anus and anal canal | 19 | 0.43% |
| **Total** |  | 4386 | 100.00% |
| **Metastatic sites** | | (N = 4386) |  |
| **C78.7** | Secondary malignant neoplasm of the liver | 1937 | 44.16% |
| **C78.0** | Secondary malignant neoplasm of the lung | 1061 | 24.19% |
| **C79.5** | Secondary malignant neoplasm of bone and bone marrow | 72 | 1.64% |
| **C78.6** | Secondary malignant neoplasm of retroperitoneum and peritoneum | 65 | 1.48% |
| **C78.8** | Secondary malignant neoplasm of other and unspecified digestive organs | 58 | 1.32% |
| **C79.3** | Secondary malignant neoplasm of brain and cerebral meninges | 43 | 0.98% |
| **C79.1** | Secondary malignant neoplasm of bladder and other and unspecified urinary organs | 14 | 0.32% |
| **C79.6** | Secondary malignant neoplasm of ovary | 6 | 0.14% |
| **C78.2** | Secondary malignant neoplasm of pleura | 4 | 0.09% |
| **C79.0** | Secondary malignant neoplasm of kidney and renal pelvis | 3 | 0.07% |
| **C79.2** | Secondary malignant neoplasm of skin | 3 | 0.07% |
| **C78.3** | Secondary malignant neoplasm of other and unspecified respiratory organs | 1 | 0.02% |
| **C79.7** | Secondary malignant neoplasm of adrenal gland | 1 | 0.02% |
